# Implementing a multilevel intervention to accelerate colorectal cancer screening and follow-up in federally qualified health centers using a stepped wedge design: a study protocol

**DOI:** 10.1186/s13012-020-01045-4

**Published:** 2020-10-29

**Authors:** Karen Kim, Blasé Polite, Donald Hedeker, David Liebovitz, Fornessa Randal, Manasi Jayaprakash, Michael Quinn, Sang Mee Lee, Helen Lam

**Affiliations:** 1 Center for Asian Health Equity, University of Chicago, 5841 S. Maryland Ave, MC1140, Chicago, IL 60637 USA; 2 University of Chicago Medicine Hematology and Oncology, 5841 S Maryland Ave, Chicago, IL 60637 USA; 3 Department of Public Health Sciences, University of Chicago Biological Sciences, 5841 S. Maryland Ave, Rm W-254, MC2000, Chicago, IL 60637 USA; 4 Division of General Internal Medicine & Geriatrics, Feinberg School of Medicine, Northwestern University, 750 N. Lake Shore Dr., 10th Floor, Chicago, IL 60611 USA; 5 Department of Internal Medicine, Section of General Internal Medicine, University of Chicago, 5841 S Maryland Ave, Chicago, IL 60637 USA

**Keywords:** Colorectal cancer, Multilevel intervention, Stepped wedge design, Implementation strategy, Federally qualified health center, FQHC

## Abstract

**Background:**

Screening for colorectal cancer (CRC) not only detects disease early when treatment is more effective but also prevents cancer by finding and removing precancerous polyps. Because many of our nation’s most disadvantaged and vulnerable individuals obtain health care at federally qualified health centers, these centers play a significant role in increasing CRC screening among the most vulnerable populations. Furthermore, the full benefits of cancer screenings must include timely and appropriate follow-up of abnormal results. Thus, the purpose of this study is to implement a multilevel intervention to increase rates of CRC screening, follow-up, and referral-to-care in federally qualified health centers, as well as simultaneously to observe and to gather information on the implementation process to improve the adoption, implementation, and sustainment of the intervention. The multilevel intervention will target three different levels of influences: organization, provider, and individual. It will have multiple components, including provider and staff education, provider reminder, provider assessment and feedback, patient reminder, and patient navigation.

**Methods:**

This study is a multilevel, three-phase, stepped wedge cluster randomized trial with four clusters of clinics from four different FQHC systems. In the first phase, there will be a 3-month waiting period during which no intervention components will be implemented. After the 3-month waiting period, we will randomize two clusters to cross from the control to the intervention and the remaining two clusters to follow 3 months later. All clusters will stay at the same phase for 9 months, followed by a 3-month transition period, and then cross over to the next phase.

**Discussion:**

There is a pressing need to reduce disparities in CRC outcomes, especially among racial/ethnic minority populations and among populations who live in poverty. Single-level interventions are often insufficient to lead to sustainable changes. Multilevel interventions, which target two or more levels of changes, are needed to address multilevel contextual influences simultaneously. Multilevel interventions with multiple components will affect not only the desired outcomes but also each other. How to take advantage of multilevel interventions and how to implement such interventions and evaluate their effectiveness are the ultimate goals of this study.

**Trial registration:**

This protocol is registered at clinicaltrials.gov (NCT04514341) on 14 August 2020.

Contributions to the literature

- Study findings will provide the evidence base for multilevel interventions to increase rates of colorectal cancer screening and follow-up care among racial/ethnic minority and low-income populations, which disproportionately receive health care in federally qualified health centers.
- Although the parallel cluster-randomized trial is a gold standard for causal inference, it may not be feasible in real-world settings. The stepped wedge design retains some elements of randomization, allows multiple data collection points, and enhances the precision of the study. This study will provide evidence of the flexibility and feasibility of a stepped wedge design in multilevel interventional studies.
- The effectiveness of an intervention, in part, depends on the strength of the implementation process. This study will demonstrate how to use the Consolidated Framework for Implementation Research (CFIR) to conduct an ongoing evaluation of the implementation process.

## Background

### Colorectal cancer control and safety-net health systems

Colorectal cancer is the second leading cause of cancer death in the United States [1]. Screening for CRC not only detects disease early when treatment is more effective but also prevents cancer by finding and removing precancerous polyps. Although CRC screening is effective in preventing and reducing CRC mortality, it remains underutilized. Despite strong evidence to support CRC screening, nationally, only 68.8% of adults had up-to-date screening in 2018 [2]. While the percentage of age-eligible adults who are up-to-date with recommended CRC screening has been increasing over the years, nearly 28% of the eligible adults have never been screened, and this figure is even higher among racial/ethnic minorities and people who live in poverty [3–6].

Individuals who are without health insurance or a regular care provider are more likely to have never been screened than those with health insurance and a regular care provider [3]. Racial/ethnic minorities and people with low social-economic status (SES) are often among those who lack health insurance and a regular source of care. Therefore, the underuse of CRC screening is more frequent among these populations [4–6]. These populations also disproportionately receive health care in safety-net settings, such as federally qualified health centers (FQHCs) [7, 8]. FQHCs are designed to provide comprehensive, quality primary healthcare services to medically underserved communities and vulnerable populations. In 2018, FQHCs served 28 million patients, of whom 23% were uninsured, 63% were racial/ethnic minorities, and 91% were living below 200% poverty level [9]. Because many of our nation’s most disadvantaged and vulnerable individuals obtain health care at FQHCs, these health centers play a significant role in increasing CRC screening among the most vulnerable populations.

### Challenges of colorectal cancer control in safety-net healthcare systems

Due to the cost and limited availability of specialty services combined with patient preferences, safety-net settings often promote non-invasive screening methods, such as fecal occult blood test (FOBT) or fecal immunochemical tests (FIT), as the modality for screening [10–13]. To achieve the benefits of CRC screening using FOBT/FIT, timely follow-up of positive results must occur. Failure to offer or complete a diagnostic evaluation following a positive FOBT/FIT has several consequences, including late-stage CRC diagnoses. This failure could undermine the benefits of screening and increase disparities [14–17]. While no national estimates of the proportion of individuals without follow-up diagnostic evaluation after receiving a positive FOBT/FIT exist, several studies report follow-up rates ranging from less than 50 to 90% within 1 year of a positive test [18–27]. Furthermore, follow-up rates varied across healthcare systems, with integrated healthcare systems exhibiting higher follow-up rates by 12 months (82–86%) compared with safety-net health systems (56–58%) [22, 28–30]. Safety-net healthcare systems not only face challenges to increase CRC screening rates among racial/ethnic minorities and low SES patients but also to increase follow-up diagnostic evaluation rates after a positive FOBT/FIT.

### Multilevel influences on cancer screening behavior

**Fig. 1:**
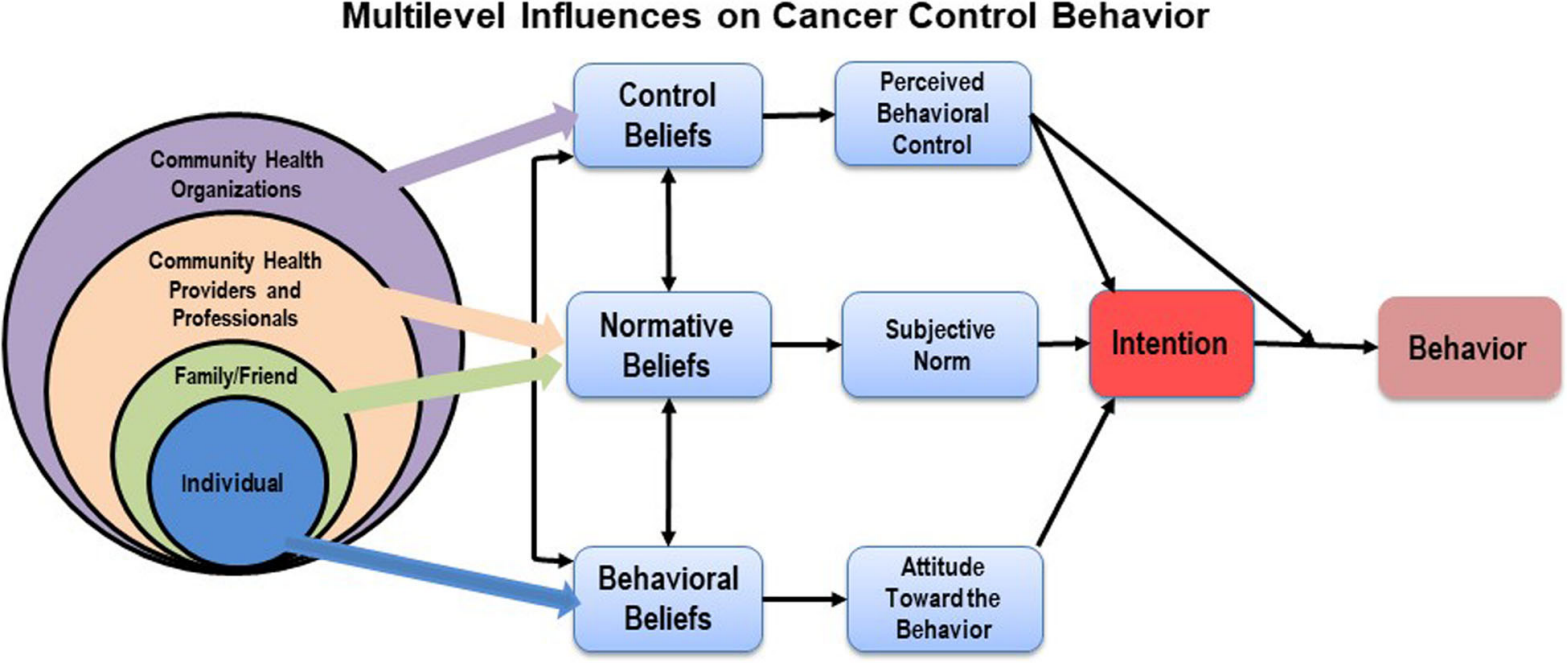
Multilevel influences on cancer control behavior

Individuals live and seek care in a complex environment, and multiple levels of contextual influences may affect individual decisions and actions [31, 32]. Traditionally, behavior change models, such as the Theory of Planned Behavior Model (TPB), mainly focus on the individual and do not explicitly show how contextual factors may affect and interact with the individual. By incorporating system models, such as the Social Ecological Model, into an individual behavior change model, we can then expand such models to consider multilevel contextual influences, which affect the individual through interdependent interaction [33]. Figure 1 shows the integrated Social Ecological Model and the TPB Model, which illustrates a more holistic review of cancer screening behavior. Multilevel interventions target changes in more than one contextual level (e.g., organization, provider, and patient levels) to influence health behavior, healthcare practice, and health outcomes [34–36]. Although the call for multilevel interventions has increased [36–39], there is still a lack of evidence addressing how to implement multilevel interventions or how interventions at multiple levels interact and affect health outcomes.

### Aims and objectives

ACCSIS-Chicago (Accelerating Colorectal Cancer Screening and Follow-up Through Implementation Science – Chicago) is a 4-year study. The overall objective of this study is to provide the evidence base for multilevel interventions that increase rates of CRC screening, follow-up, and referral-to-care at federally qualified health centers, and to understand how to improve the adoption, implementation, and sustainment of these interventions. This study is built on the lessons learned from the 1-year pilot feasibility study and our 5-year CDC funded Colorectal Cancer Control and Prevention Screening Program that used a multilevel and organized approach to increase CRC screening rates among integrated healthcare systems and FQHCs. Although we obtained programmatic success in increasing CRC screening rates with our CDC funded project among our partner health systems, the process to achieve these positive outcomes and how multilevel components interact and influence each other remained unknown. In this study, we will test the effectiveness of our multilevel intervention while simultaneously observing and gathering information on the implementation process. Furthermore, we will examine how intervention components at different levels interact and influence health outcomes. The selection of our multilevel intervention components is based on extensive literature review, the strength of evidence, and findings from our previous studies and projects. The multilevel intervention will target three different levels of influence (organization, provider, and individual) to improve rates of CRC screening, follow-up, and referral-to-care at our partner FQHCs. Our multilevel intervention will have multiple components, including provider and staff education, provider reminder, provider assessment and feedback, patient reminder, and patient navigation.

The aims of ACCSIS-Chicago are fourfold: (1) use a stepped wedge design to implement a multilevel intervention in three phases, (2) collect quarterly data to track changes over time, (3) evaluate the implementation process and the effectiveness of implementation strategies through observations, interviews, and annual survey, and (4) evaluate the effectiveness of the multilevel intervention using multilevel and longitudinal modeling.

## Methods

This study is conducted as part of the NCI-funded consortium, The Accelerating CRC Screening and Follow-up through Implementation (ACCSIS) Program. The overall aim of ACCSIS is to conduct multi-site, coordinated, transdisciplinary research to evaluate and improve CRC screening processes using implementation science strategies. The ACCSIS-Chicago will implement a multilevel, multicomponent intervention to increase rates of CRC screening, follow-up, and referral-to-care at four federally qualified health systems located in Illinois and Indiana. The protocol has been reviewed and approved by the University of Chicago Institutional Review Board (IRB19-1496) and is registered at clinicaltrials.gov (NCT04514341).

### Conceptual framework

**Fig. 2:**
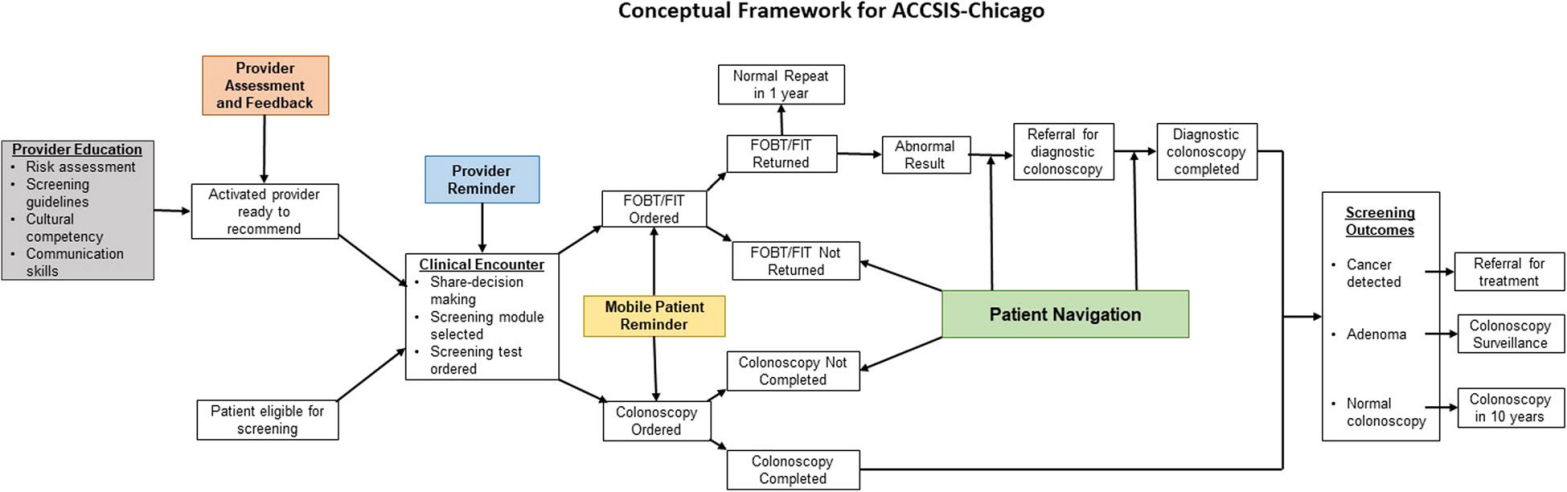
Conceptual framework for ACCSIS–Chicago

CRC screening and follow-up processes are complex and include several steps and interfaces. However, very few interventional studies have simultaneously targeted patient-, provider-, and organization-level factors. Interventions that focus on reducing barriers across several levels will likely be more effective for increasing rates of CRC screening, follow-up, and referral-to-care. Figure 2 shows the conceptual framework for ACCSIS-Chicago and how the multilevel components work together along the CRC control and prevention continuum to achieve the desired outcomes.

### Multilevel intervention and implementation strategy

#### Provider level component

##### Provider education

Numerous studies indicate the importance of physician recommendation in influencing a patient’s CRC screening decision [40–53], and this factor is a primary predictor for patient adherence with screening guidelines [54–56]. Provider challenges for CRC screening include (1) asymptomatic patients believe that screening is unnecessary, especially when offered an invasive procedure, and (2) the different screening methods may cause confusion, which underscores the scientific ambiguity surrounding the decision [49]. Studies have shown that shared decision-making can improve patient compliance when decisions are complex, such as CRC screening [57–59], yet many clinicians lack training in shared decision-making [60]. They are often uncertain about which decisions require patient participation, and about how to communicate technical concepts to patients in simple language that is accurate, balanced, and understandable. Thus, provider education is an important component for improving CRC screening compliance. The research team will provide tailored education and guidance on best practices for participating health professionals. The educational sessions will focus on CRC risk assessment, screening guidelines, cultural competency, shared decision-making, and communication skills using an academic detailing approach. The academic detailing approach involves trained experts visiting healthcare professionals in their settings to provide tailored education and guidance on best practices, which have shown to have a significant effect on increasing rates of CRC screening [61–66]. Each session will last about 15 to 20 min and will take place during routine staff and provider meetings. These academic detailing sessions will be ongoing throughout the study period.

##### Provider assessment and feedback

We will combine academic detailing with practice facilitation, which includes assessment and feedback on aggregate and individual provider screening behavior and practice performance [67]. We will capture data from the electronic health record (EHR) across our partner sites to generate clinic- and provider-specific reports. The reports will include CRC screening order rates, screening completion rates, follow-up rates for abnormal results, and referral to oncology care. Providers will be able to see each other’s performances, which allows providers to compare and learn from each other’s successes. Our research team will deliver the feedback reports quarterly.

##### Provider reminder

As previously mentioned, studies have shown that provider recommendations have the most significant impact on CRC screening rates. However, given the multitude of competing priorities during a patient encounter, CRC screening recommendations can be overlooked. A provider prompt generated electronically or manually by staff members will be implemented to remind providers to screen their eligible patients. Provider prompts have been shown to increase CRC screening rates [68].

#### Organization and individual level component

##### Patient reminder

Provider recommendation alone does not guarantee the completion of CRC screening, which involves patient compliance. We will implement a patient reminder component using a text-based platform, also known as short message service (SMS), to engage and remind patients to complete the screening. Patients who use a stool-based screening method will receive an initial text message with links to videos and educational materials within 3 to 5 days after the ordered test. Patients will continue to receive text messages weekly for 90 days as a reminder to complete and return the stool card for testing. When patients respond that they have returned the stool card, or when they opt out of the reminder system, the SMS will stop. For patients who use colonoscopy as a screening method, the SMS will also include instruction on bowel preparation and dietary restrictions 5 days before the procedure. Studies have demonstrated that SMS patient reminders not only had a positive impact on screening rates but also improved the quality of bowel preparation [69–74].

##### Patient navigation

Patient navigation services will address both organization and individual influences. Patient navigation focuses on eliminating barriers by guiding a patient through a complex healthcare system, addressing education, sociocultural, and logistical needs using trained staff. Patient navigation has consistently shown an association with increased CRC screening completion [75–82] and diagnostic follow-up after a positive test result [83–87]. We will hire and train two full-time CRC navigators and adapt and modify the well-studied New Hampshire Colorectal Cancer Screening Program (NHCRCSP) Patient Navigator Model [88–90]. Navigator training will include didactic and clinical sessions with ongoing training every quarter throughout the duration of the program. CRC Navigators will have field training at each healthcare system to become familiar with the clinic workflow and referral process, as well as an opportunity for clinical shadowing in the CRC oncology clinic and colonoscopy suite. CRC navigators will also undergo pre and post-training evaluation, and they will be centralized and supervised by the project director. All CRC navigators will meet biweekly to discuss their cases, share information, and address any issues associated with their roles and responsibilities.

### Implementation strategy

The effectiveness of an intervention, in part, depends on the strength of the implementation process. A successful implementation process requires attention to critical contextual factors. From the very outset, we will conduct a pre-implementation organizational readiness assessment (ORA). We will use the CFIR framework (Consolidated Framework for Implementation Research) to guide our ORA. CFIR is a conceptual framework that was developed to conduct a systematic assessment of multilevel implementation contexts to identify factors that might influence intervention implementation and effectiveness [91]. The CFIR framework consists of five domains (intervention characteristics, inner setting, outer setting, characteristics of individuals, and processes) and a standard set of constructs that allows a comprehensive assessment. We will conduct key informant interviews with health system leadership and clinic leaders to evaluate: (1) current CRC screening workflow, (2) CRC data capturing and validation process, and (3) capacity and resources available to support the implementation. Table 1 summarizes the interview questions based on CFIR domains. Also, we will conduct a readiness survey with clinical providers and support staff to assess organizational climate and culture. Our 29 survey items were adapted and modified from validated instruments that measured CFIR constructs using a Delphi method [92–95].

**Table 1:**
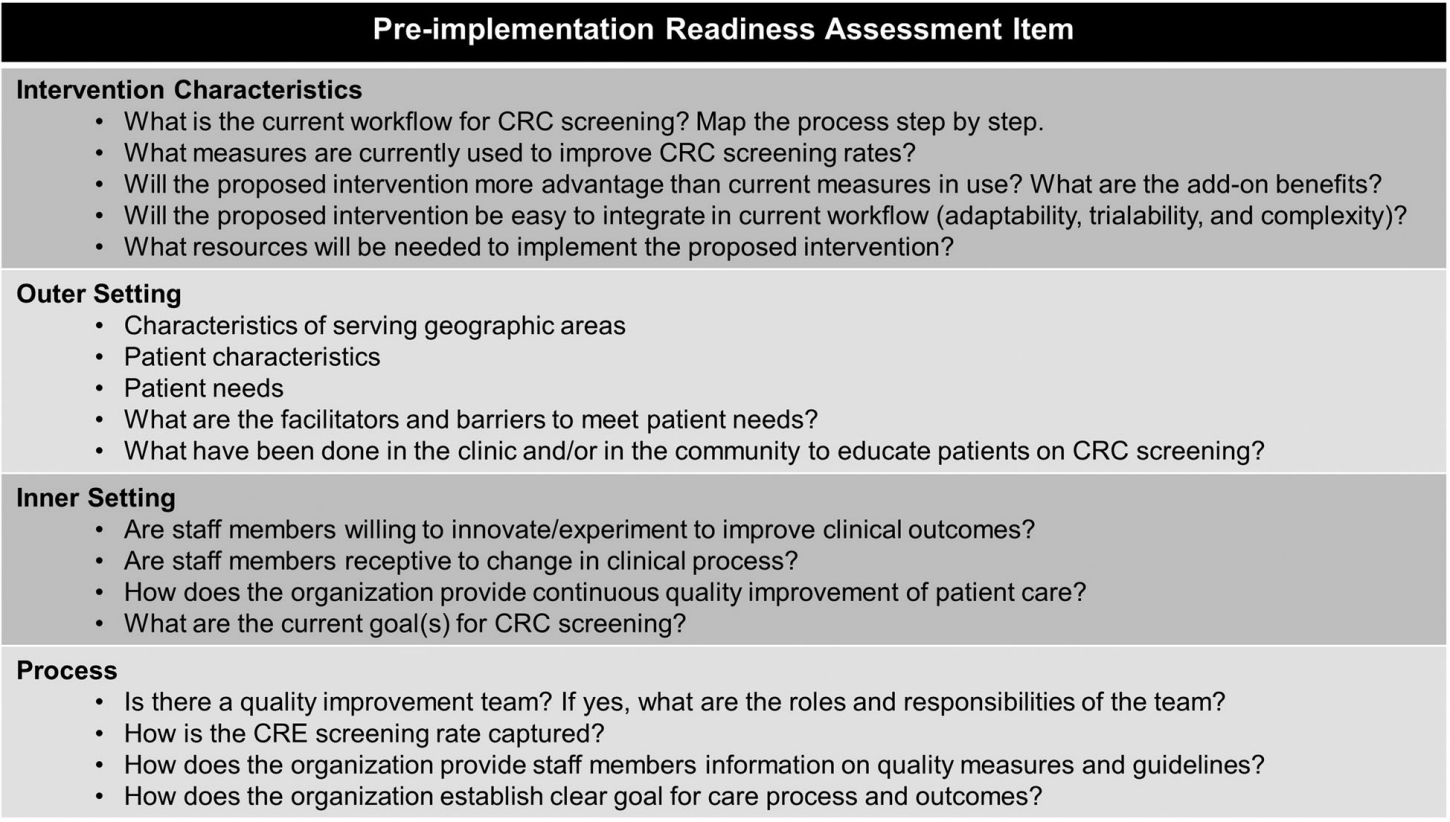
Pre-implementation readiness assessment guided by CFIR domains

Effective strategies to support the implementation process is critical. Implementation strategies are actions taken to enhance the adoption, implementation, and sustainability of evidence-based interventions. Table 2 summarizes the implementation strategies that we will use to implement our multilevel intervention.

**Table 2:**
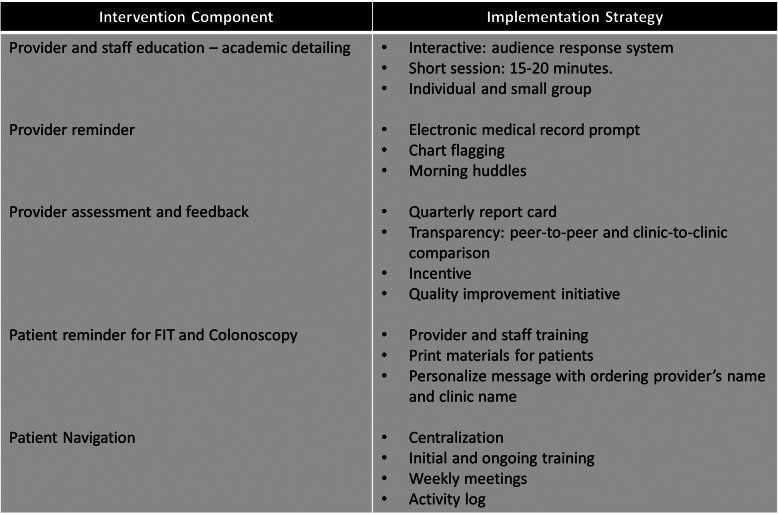
Implementation strategies for each intervention component

**Fig. 3:**
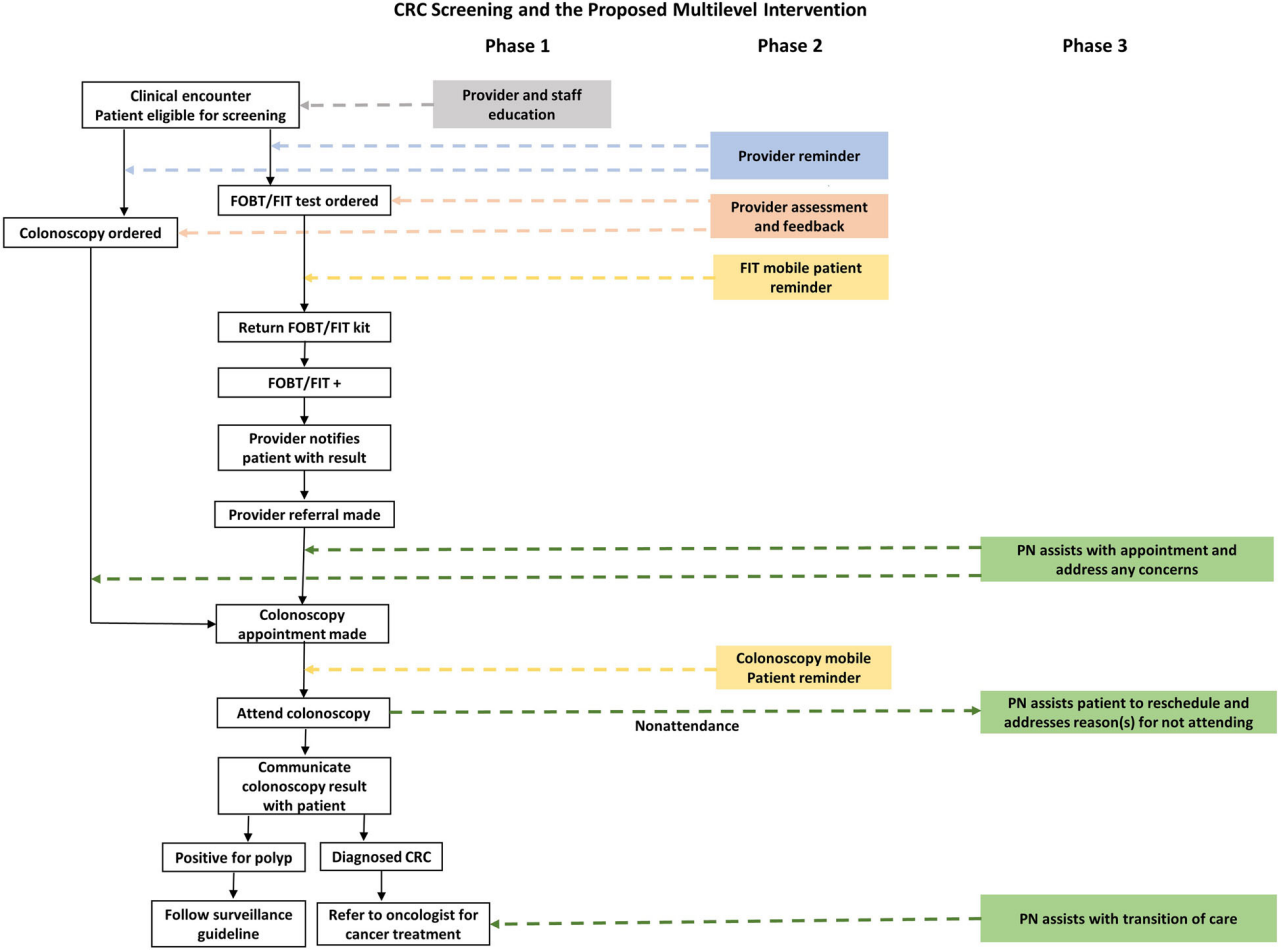
CRC screening and the proposed multilevel intervention

Finally, we will implement our multilevel, multicomponent intervention in three phases (Fig. 3) along the CRC screening continuum.

### Study design

In the evaluation of a multilevel intervention, an important design decision is whether to assess only the combined impact of the multiple components compared to no intervention or the separate effects of each component at different levels and the possible interaction effects [96]. While a parallel cluster randomized trial is a gold standard for causal inference, it requires a large number of clusters to assess separate effects of a multilevel intervention, which may not be feasible in real-world settings. To overcome this issue and ensure the standard of rigorous scientific evaluation, we will use a stepped wedge cluster randomized trial design to evaluate the effectiveness and impact of our multilevel intervention. The stepped wedge is a pragmatic study design and retains some elements of randomization as it is a controlled trial [97–99]. The stepped wedge design includes an initial period of no exposure. Then one cluster (or group of clusters) will be randomized to cross from the control to the intervention at regular intervals. In the end, all clusters will be exposed to the intervention component but not at the same point in time. Since each cluster contributes to both exposed and unexposed observations and acts as its own control, such design can enhance the precision of the study [97]. Furthermore, the feature of multiple data collection points in stepped wedge design allows the researcher to capture the impact of an intervention when such impact develops over time, or when the intervention needs an initial period of adjustment before becoming embedded in the setting. This feature is especially valuable for the ACCSIS-Chicago study because we expect our outcomes will change over time rather than at a discrete-time point.

**Fig. 4:**
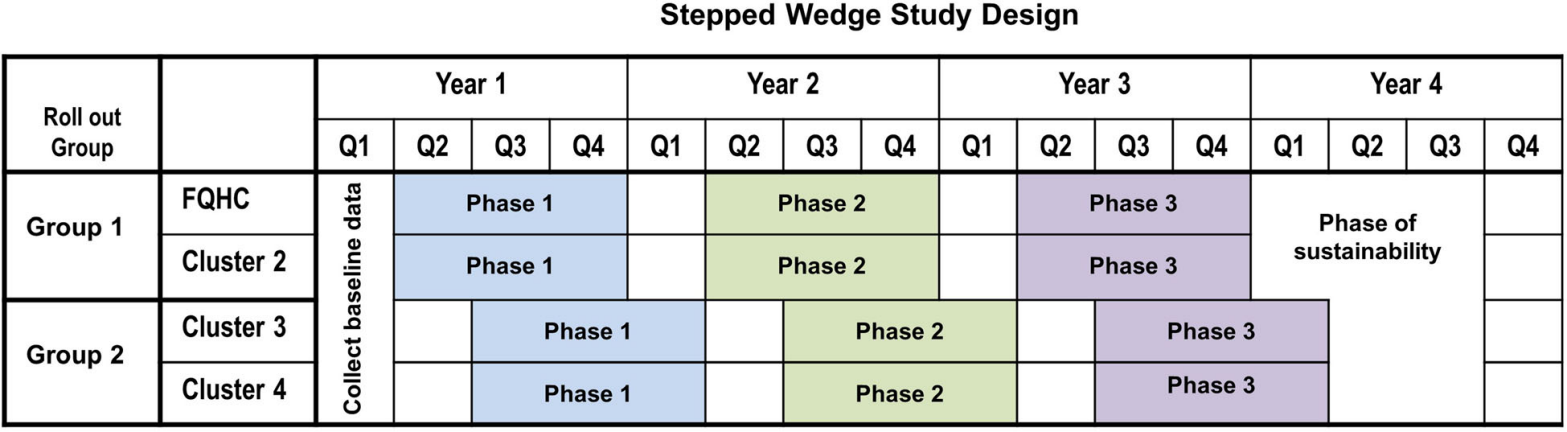
Stepped wedge study design

We will have four clusters of clinics from four different federally qualified health systems. Each cluster will have 5 to 12 clinics and a total of 20 to 35 primary care providers (internists, family practice physicians, nurse practitioners, and physician assistants). Our biostatistician will conduct the randomization independently from the research team. Figure 4 shows the stepped wedge study design. The study design will include an initial 3-month period during which no intervention components will be implemented. We will use this 3-month waiting period to collect baseline outcome data from the previous year. After the 3-month waiting period, we will randomize two clusters to cross from the control to the intervention and the remaining clusters to follow 3 months later. All clusters will stay at the same phase for 9 months, followed by a 3-month transition period, and then cross over to the next phase. We will collect quarterly data throughout the 4-year study period.

### Study sites

We will partner with four FQHCs, two in Illinois and two in Indiana. Together, our FQHC partners have 40 primary care clinics and 130 primary care providers and served 162,000 individual patients in 2018. Of the 162,000 patients, 78% were racial minorities, 93% live at or below 200% poverty, and 17% were uninsured. The CRC screening rates among our partners range from 25 to 43%.

### Data sources and outcome measures

During the first 6 months, we will collect baseline data on primary outcomes (Table 3) from the EHR systems. After the initiation of the implementation process, we will collect primary outcome measures each quarter until the conclusion of the study completion.

**Table 3:**
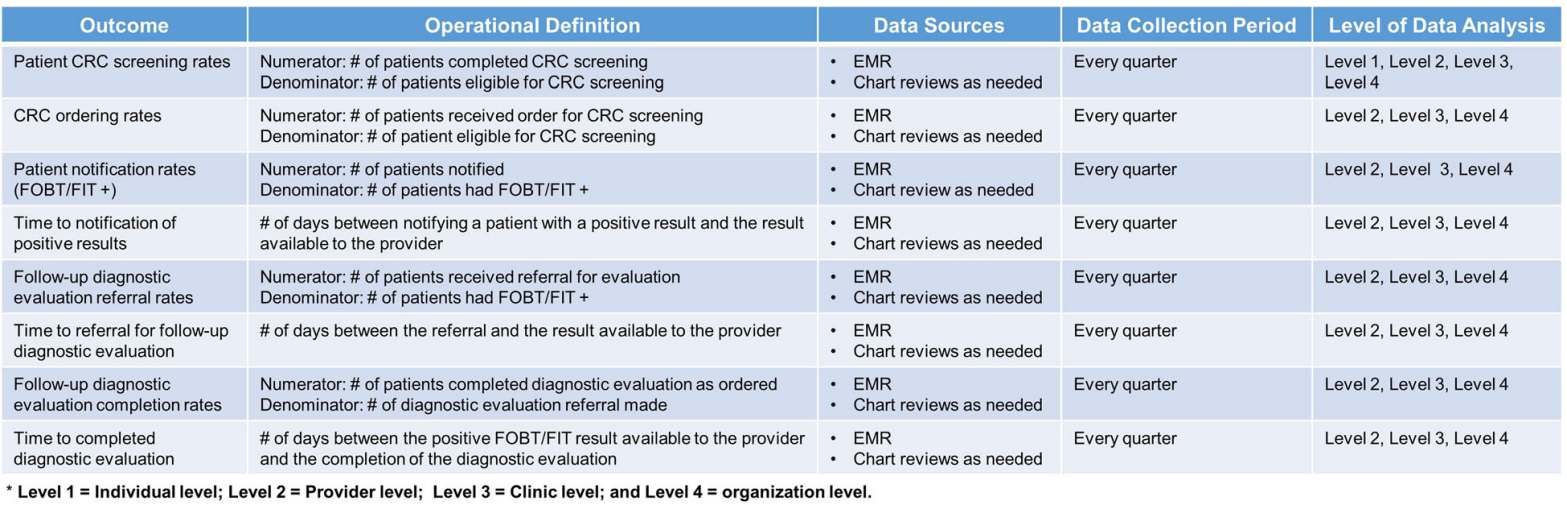
Outcome measures for the multilevel intervention

For the patient navigation component, we will also collect data from the navigator activity logs, including the number of patients being served, length of the encounter, types of encounter (e.g., transportation arrangement or making the appointment), and types of action taken. Furthermore, we will also ask patients who received navigation services to complete a patient satisfaction survey adapted from the patient satisfaction survey developed by the NCI-sponsored Patient Navigation Research Program [100].

### Process evaluation

We will assess the potential causal and contextual factors that may be associated with observed outcomes at the provider and clinic levels. After completion of each phase, we will send all providers at our partner clinics a survey to assess their exposure to and experience with the intervention component. Furthermore, we will evaluate the changes in organizational culture and climate that may ascertain potential mechanisms of impact using the 29-survey items we developed based on the CFIR.

### Data analysis plan

We will conduct descriptive analyses, as well as within-group and between-group changes over time. Furthermore, to evaluate the four-component intervention effects, we will take a step-by-step approach. First, each intervention effect at each time phase will be estimated separately using linear-mixed effects models. The estimate of intervention effect in each model will be used to test if additional intervention component significantly affects outcome measures. Next, we will develop a grand model, including all intervention components simultaneously in addition to the models described above.

In this study, patients are nested within providers, and providers are nested within clinics. The problem with nested data structures is that they violate the independence assumption of traditional regression models. Thus, we will use multilevel modeling to analyze our data. We will start with the unconditional (null) three-level random intercept models:

Level – 1: *Y*_*ijk*_ = β_0jk_ + *е*_ijk_

Level – 2: β_0*jk*_ = β_0k_ + *u*_jk_

Level – 3: β_0k_ = β_0_ + *v*_k_

where *Y*_*ijk*_ is the observed outcome for patient *i* with provider *j* in clinic *k*. β_0_ is the mean response across all clinics. “*v*_k_” is the random effect of clinic *k*, “*u*_jk_” is the random effect of provider *j* in clinic *k*, and е_ijk_ is the residual error. The random effects and residual errors are assumed to be normally distributed and independent of one another. Putting these submodels together yields the (null) 3-level model as: *Y*_*ijk*_ = β_0_ + *v*_k_ + *u*_jk_ + *е*_ijk_

Adding predictor variables to the models is straightforward. For example, the 3-level model with one predictor measured at each level is *Y*_*ijk*_ = β_0_ + β_1_X_1ijk_ + β_2_X_2jk_ + βX_3k_ + *v*_k_ + *u*_jk_ + *е*_ijk,_ where β_0_ + β_1_X_1ijk_ + β_2_X_2jk_ + βX_3k_ is the fixed effect component of the model and *v*_k_ + *u*_jk_ + *е*_ijk_ is the random effect component of the model. The fixed effect of the model specifies the overall mean relationship between the response and the predictor variables. The random effect component of the model specifies how the provider and clinic specific relationships differ from the overall mean relationship. To assess any cross-level interaction effects, we will add a product term to the model. For example, the model for a level-1 predictor (e.g., age) interacting with a level-2 predictor: *Y*_*ijk*_ = β_0_ + β_1_X_1ijk_ + β_2_X_2jk_ + β_3_ (X_1ijk_ ● X_2jk_) + *v*_k_ + *u*_jk_ + *е*_ijk._ Since one of the patient-level (level-1) outcome measures is dichotomous (whether the patient adherence to follow-up diagnostic evaluation after a positive result within 9 months or not), we will use a multilevel logistic model for binary data. In this case, the level-1 model is in terms of the logit of the response of the level-1 binary outcome *Y*_*ijk*_, for example:

Level – 1: log [*P*_ijk/_ (1 – *P*_ijk_)] = β_0jk_ + β_1_X_ijk_.

In this study, we will use the restricted maximum likelihood (REML) to estimate variances. REML treats the regression coefficients as unknown quantities to be an estimate based on sample data and subtracts the needed degree of freedom when computing variance estimates. Since REML only allows for tests of models that differ in their variances, we will calculate the intraclass correlation (ICC) to access the variation in response variable across providers and clinics before testing the models. Statistical analyses will be conducted using Statistical Package for the Social Science (SPSS) version 26.0 and Stata version 15, with the significance level set at alpha ≤0.05.

### Sample size and power

Although we will implement the multilevel intervention over three phases, the power of the study is calculated based on the phase 1 period only. In our pilot study, the baseline rate in the intervention group was 30.8%, and it went up to 40.7% in the year 2019. We observed the intra-class correlation as 0.135. Considering four components of the intervention, we lower the significance level to 0.05/4 = 0.017. We will recruit a total of 40 clinics and 130 providers across four partner healthcare systems (clusters) (see Table 3). Although the number of clinics and providers varies between clusters, we calculate the power based on the average number of providers, which is 32 providers per cluster. Under these conditions, the study will have power in excess of 90% to detect an increase in the CRC screening rate of 32%. Table 4 shows the levels of powers for different sample sizes and effect sizes for reference.

**Table 4:**
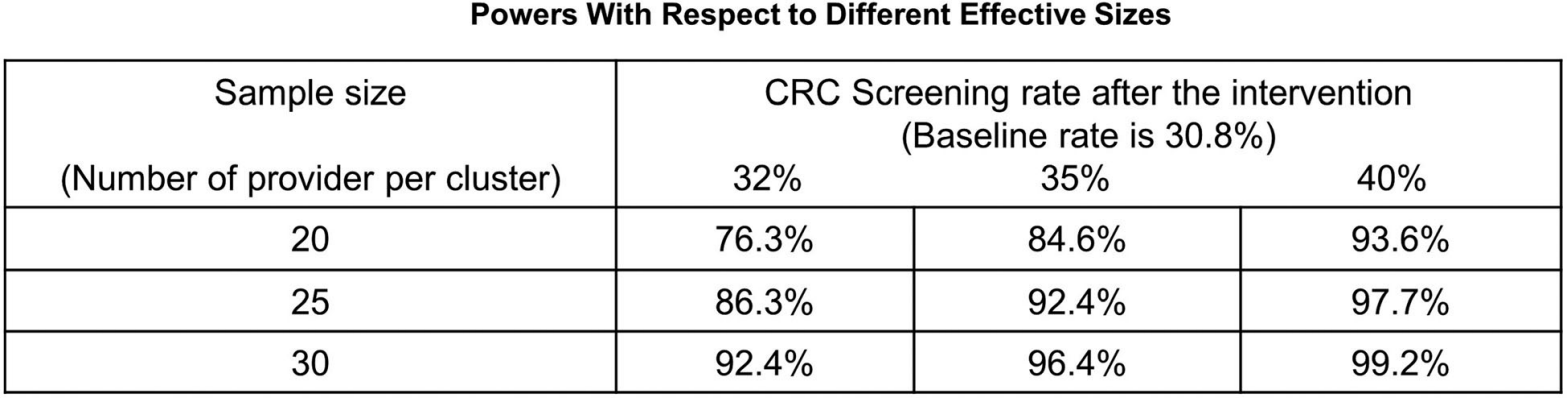
Powers with respect to different effective sizes

## Discussion

### Limitations and related considerations

#### Using EHR data for research

The adoption of electronic health records (EHRs) has significantly increased the amount of detailed patient health information available today, which would be difficult to obtain using survey data alone. Using data collected in EHRs can improve clinical research efficiency, including the investigation of relationships between interventions and outcomes [101, 102]. However, raw EHR data are disorganized and full of uncodified variables. There remain immense hurdles to extract and apply EHR data effectively for research purposes. Furthermore, our partners use different EHR systems, including Epic, NextGen, and Athena. We have learned valuable techniques and steps to capture data from different EHR systems in our previous studies. We will work with our health system partners to develop a plan for data capturing and train a data coordinator at each site to effectively extract the required data and locate other data sources that may fill the gaps. Furthermore, we will implement a data validation process by randomly selecting 5% of the medical records and manually reviewed to ensure the integrity and quality of the data.

#### Evaluating the impact on follow-up diagnostic colonoscopy

Only about 4% to 6% of patients who use fecal occult blood test (FOBT) or fecal immunochemical test (FIT) as a screening modality will have a positive result and need follow-up diagnostic evaluation. The success of this study to measure improvement in follow-up rates will depend on the screening rates using FIT/FOBT among our partner FQHCs. To address this potential pitfall, we will continue to monitor the CRC screening rates among our partner FQHCs and the use of FIT/FOBT as a screening modality.

### Potential for impact and implications and plan for dissemination

There is a pressing need to reduce disparities in CRC outcomes, especially among racial/ethnic minority populations and among populations who live in poverty. There is also a need to understand the design and implementation of pragmatic studies in real-world settings within healthcare systems. Single-level interventions are often insufficient to lead to sustainable changes. Multilevel interventions, those that target two or more levels of changes, are needed to address multilevel influences simultaneously. Multilevel interventions with multiple components will affect not only the desired outcomes but also each other. How to take advantage of multilevel interventions and how to implement such interventions and evaluate their effectiveness are the ultimate goals of this study. ACCSIS-Chicago will provide the needed evidence for future multilevel interventional studies, especially in increasing CRC screening, follow-up, and referral-to-care. Furthermore, it will demonstrate best practice for how multilevel interventions can be scaled-up nationwide to reduce the burden of CRC among racial/ethnic and low-income populations that usually seek health care in resource strained safety-net systems.

The rigor, design, feasibility, and high likelihood of success of this study will provide crucial evidence regarding multilevel interventions that target three levels of influences (organization, provider, and individual) to improve CRC screening, follow-up, and referral-to-care. We will disseminate these findings through peer-reviewed publications, presentations, and professional meetings. Throughout this effort, we will share data following the National Cancer Institute NCI) policies and submit standard data elements to the NCI data repository.

## Data Availability

Not applicable.

## Abbreviations

CRC: Colorectal cancer
SES: Low social-economic status
FQHC: Federally qualified health center
FOBT: Fecal occult blood test
FIT: Fecal immunochemical test
TPB: Theory of Planned Behavior Model
ACCSIS-Chicago: Accelerating Colorectal Cancer Screening and Follow-Up Through Implementation Science – Chicago
CDC: Center for Disease Control and Prevention
EHR: Electronic health record
SMS: Short message service
ORA: Organization readiness assessment
CFIR: Consolidated Framework for Implementation Research
